# Identification and replication of RNA-Seq gene network modules associated with depression severity

**DOI:** 10.1038/s41398-018-0234-3

**Published:** 2018-09-05

**Authors:** Trang T. Le, Jonathan Savitz, Hideo Suzuki, Masaya Misaki, T. Kent Teague, Bill C. White, Julie H. Marino, Graham Wiley, Patrick M. Gaffney, Wayne C. Drevets, Brett A. McKinney, Jerzy Bodurka

**Affiliations:** 1 Department of Mathematics, The University of Tulsa, Tulsa, OK USA; 2 Laureate Institute for Brain Research, Tulsa, OK USA; 3 School of Community Medicine, University of Tulsa, Tulsa, OK USA; 4 Department of Educational Psychology, University of Nebraska-Lincoln, Lincoln, NE USA; 5 Departments of Surgery and Psychiatry, University of Oklahoma School of Community Medicine, Tulsa, OK USA; 6 Department of Pharmaceutical Sciences, University of Oklahoma College of Pharmacy, Tulsa, OK USA; 7 Department of Biochemistry and Microbiology, Oklahoma State University Center for the Health Sciences, Tulsa, OK USA; 8 Tandy School of Computer Sciences, The University of Tulsa, Tulsa, OK USA; 9 Department of Surgery, Integrative Immunology Center, University of Oklahoma School of Community Medicine, Tulsa, OK USA; 10 Arthritis and Clinical Immunology Research Program, Division of Genomics and Data Sciences, Oklahoma Medical Research Foundation, Oklahoma City, OK USA; 11 Janssen Research & Development, LLC, Johnson & Johnson, Inc, Titusville, NJ USA; 12 Stephenson School of Biomedical Engineering, University of Oklahoma, Norman, OK USA

**Keywords:** Depression, Predictive markers

## Abstract

Genomic variation underlying major depressive disorder (MDD) likely involves the interaction and regulation of multiple genes in a network. Data-driven co-expression network module inference has the potential to account for variation within regulatory networks, reduce the dimensionality of RNA-Seq data, and detect significant gene-expression modules associated with depression severity. We performed an RNA-Seq gene co-expression network analysis of mRNA data obtained from the peripheral blood mononuclear cells of unmedicated MDD (*n* = 78) and healthy control (*n* = 79) subjects. Across the combined MDD and HC groups, we assigned genes into modules using hierarchical clustering with a dynamic tree cut method and projected the expression data onto a lower-dimensional module space by computing the single-sample gene set enrichment score of each module. We tested the single-sample scores of each module for association with levels of depression severity measured by the Montgomery-Åsberg Depression Scale (MADRS). Independent of MDD status, we identified 23 gene modules from the co-expression network. Two modules were significantly associated with the MADRS score after multiple comparison adjustment (adjusted *p* = 0.009, 0.028 at 0.05 FDR threshold), and one of these modules replicated in a previous RNA-Seq study of MDD (*p* = 0.03). The two MADRS-associated modules contain genes previously implicated in mood disorders and show enrichment of apoptosis and B cell receptor signaling. The genes in these modules show a correlation between network centrality and univariate association with depression, suggesting that intramodular hub genes are more likely to be related to MDD compared to other genes in a module.

## Introduction

RNA-Seq is a transcriptome profiling technique that uses next-generation sequencing to provide a sensitive, quantitative measurement of RNA abundance or gene expression. Challenges associated with the RNA-Seq approach include both technical limitations (e.g., tissue heterogeneity and batch effects) and statistical concerns (e.g., over dispersion and multiple hypothesis testing). Furthermore, major depressive disorder (MDD) is a complex phenotype involving systems of interacting genes, and single-gene associations of expression have not reached genome-wide significance. While these approaches have provided biological insights and identified candidate biomarkers associated with some neurological diseases^1,2^, network and gene–gene interaction approaches may enrich the variable space to better predict or characterize the genomic architecture of more complex phenotypes^3^.

Co-expression network techniques for detecting coordinated gene expression changes at a gene set (modular) level have potential power to provide novel insights into the genetic architecture of psychiatric disorders^4,5^. A module can be understood as a collection of genes that are highly interconnected (e.g., by co-expression) and, thus, more likely to share a similar biological function^6^. Modular analysis also helps alleviate the multiple hypothesis testing problem inherent in RNA-Seq data and may be more robust than single-gene investigation. Clustering thousands of genes into pathway-sized modules and collapsing these genes onto single statistical units significantly reduces the number of hypotheses to be tested. Combined with statistical learning methods, as well as meta-analyses of existing databases, modular analyses of co-expression networks have been conducted in several studies to identify groups of differentially expressed genes in schizophrenia^7–9^, autistic spectrum disorder^10^, Alzheimer’s disease^11^, and MDD^12^.

Initial genome-wide association studies (GWAS) of MDD had limited success at finding significant variants due to the contribution of many loci with small effect sizes as well as the heterogeneous characteristics of MDD and the complex interaction between genetic variation and environmental factors^13^. More recently, many small, but significant, main effect loci have been identified through the accumulation of extremely large samples^14,15^. Similarly, it has been difficult to identify significant single-gene effects at the expression level from RNA-Seq of MDD. In Mostafavi’s RNA-Seq study of 922 subjects, only 29 genes were found to have significant association with MDD status at the relaxed FDR threshold of 0.25, but sets of top genes were significantly enriched for the IFN α/β signaling pathway^16^. Combining a modular approach with meta-analysis of 11 transcriptome studies of postmortem brains, Chang et al. identified a transcriptome module of 88 genes based on consistency with GWAS results for MDD, other neuropsychiatric disorders, and brain function^12^. This meta-module is enriched for genes that encode proteins implicated in neuronal signaling and structure.

Modularity is a ubiquitous feature of biological systems^17^ and genes within modules tend to be functionally related^18,19^, which may help us find regulatory genes that affect disease risk along with direct disease-related genes. Thus, in the present study, we applied a data-driven approach to detect depression gene modules (DGMs), which are co-expression modules associated with depression phenotypes. Because our analytical approach is sensitive to weak individual effects and takes biological interactions among genes into account, it can potentially reveal biological effects that are neglected in univariate models. Using RNA-Seq from peripheral blood, we constructed a co-expression network for the combined MDD and healthy control (HC) subjects and created hierarchical clusters of similar size using the dynamic tree cut from the weighted gene co-expression networks analysis (WGCNA) tool^20^. We then projected each subject’s gene-level expression onto a lower-dimensional space of modules using single-sample gene set enrichment analysis (ssGSEA) approach^21,22^.

The resulting 23 modular expression profiles were not conditioned on the phenotype and thus may be used as predictor variables in a greatly reduced hypothesis space. We applied false discovery rate adjusted linear regressions to each modular expression profile to identify modules that are associated with subject’s depression severity characterized by the Montgomery-Åsberg Depression Scale (MADRS). We then explored the relation of several genes in these significant modules to the clinical phenotype, MDD, along with other psychiatric disorders based on the extant literature. Of the two modular expression profiles that survived multiple hypothesis testing, one module (DGM-5) replicated in an independent data set.

## Methods and materials

**Fig. 1:**
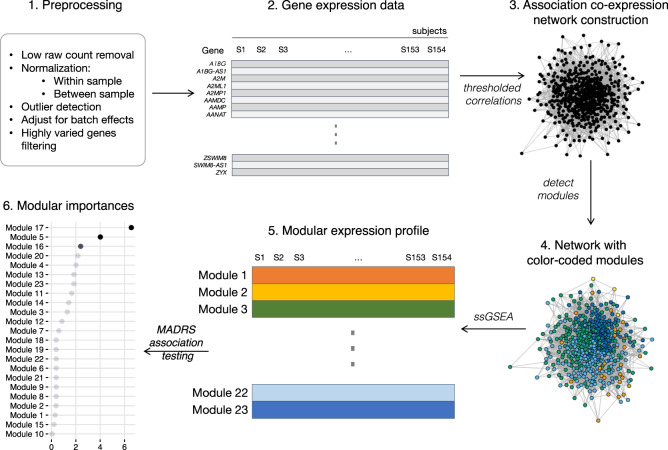
Workflow for RNA-Seq computational analyses: Preprocess the raw counts data (Step 1). Obtain normalized RNA-Seq expression values and perform coefficient of variation filtering (COV threshold = 0.8) (Step 2). Create weighted co-expression matrix and apply hard threshold (0.2) to construct an un-weighted co-expression network from the topological overlap matrix (Step 3). Detect modules using dynamic tree cut with WGCNA (Step 4). Steps (3) and (4) are iterated to tune hard threshold (0.2) to yield modules of similar size. Collapse expression of individual genes onto modules with ssGSEA (Step 5). Perform statistical testing with false discovery adjustment to find association between modules and MADRS score (Step 6). Modules passing the false discovery threshold are tested for replication in an independent study

The co-expression network module analysis involves multiple steps to obtain gene set predictors of MDD (Steps 1–6, Fig. 1). In this section, we provide details of the RNA-Seq preprocessing of the raw count data, normalization of expression values, and variation filtering (Steps 1 and 2). We describe our iterative approach to module construction using a hard threshold of the co-expression matrix and the topological overlap matrix (Step 3), combined with clustering by the dynamic tree cut algorithm (Step 4). Steps 3 and 4 are iterated with a grid of hard thresholds to obtain modules of similar size. We reduce the hypothesis space by collapsing the expression of individual genes onto these modules (Step 6) and test these module features for association with MADRS score with false discovery rate adjustment.

### Subjects

Participants between the ages of 18 and 55 years were recruited from the clinical services of the Laureate Psychiatric Clinic and Hospital (LPCH) and media advertisements in the Tulsa metropolitan area, Oklahoma. A total of *N* = 160 subjects, including 80 subjects who met DSM-IV-TR criteria for MDD (52 females, mean age = 33 ± 11) and 80 HCs who showed no history of any major psychiatric disorder in a first-degree relative (41 females, mean age = 31 ± 10), participated in the study. However, because one MDD subject’s expression data were corrupted and two additional subjects (one MDD and one HC) were outliers, their data were excluded from the analyses (see below for details). The diagnosis of MDD was established using the Structural Clinical Interview for DSM-IV-TR Axis I Disorders (SCID-I/NP; 1 January 2010) and confirmed by an unstructured interview with a psychiatrist. Exclusion criteria included the use of psychotropic medications for at least 3 weeks prior to study entry, major medical or neurological illness, psychosis, traumatic brain injury, and a history of drug/alcohol abuse within 1 year. All subjects gave written informed consent to participate in our study and received financial compensation.

The present study was approved by the Western Institutional Review Board, and it was conducted according to the principles expressed in Declaration of Helsinki. All participants gave written informed consent to participate and received financial compensation.

### Materials

The clinician-administered Montgomery-Åsberg Depression Rating Scale (MADRS; Williams & Kobak, 2008) was used to rate the severity of depressive symptoms. In clinical trials of major depressive disorder, the ten-item diagnostic questionnaire MADRS is accepted by the FDA and other health authorities as valid and reliable rating instruments for obtaining the primary outcome measure of antidepressant treatment efficacy^23^.

### Steps 1 and 2. RNA-Seq data generation and processing

Morning blood samples were obtained from the participants, and peripheral blood mononuclear cells (PBMCs) were isolated using cell preparation tubes. We quantified RNA expression obtained from frozen (PBMCs) by analyzing complementary DNA derived from the PBMCs with RNA-Seq. Following initial quality-control steps, sequencing libraries were generated using the Illumina Truseq Stranded mRNA with library prep kit according to the manufacturer’s protocol. Sequencing was performed on an Illumina Hiseq 3000 instrument with paired-end 150 bp reads. Samples were sequenced to an average depth of 30 million reads and RNA integrity number of 8.6 per sample. RNA-Seq measures gene expression by sequencing, yielding the abundance of each transcript present. After gene-level transcripts were computed from transcriptomic sequencing, the sequencing reads are aligned and mapped to individual exons. We used RefSeq for the cDNA alignment. The total number of read counts was obtained per gene from the mRNA expression. Normalization of the gene counts was performed with conditional quantile normalization (CQN), which accounts for differences in library size and also adjusts for GC content and gene length^24^. These normalized values were used for subsequent analyses. The RNA-Seq raw counts preprocessing steps involved: (i) removal of genes with low counts (threshold defined below) and normalization, (ii) outlier detection, (iii) batch effect correction, and (iv) high coefficient of variation (COV) filtering of genes. Briefly, analyses included autosomal genes with ≥15 individuals with ≥2–7 reads, depending on the library size. Then, we applied an angle-based outlier (ABO) detection^25^ to remove samples with exceptionally small ABO factor. Batch effect was adjusted with the function “removeBatchEffect” from the R package “limma”^26^ (Fig. S2). Reasoning that expression values that differ greatly across subjects are likely due to technical variability^27^, we excluded genes with (COV) larger than 0.8 to obtain genes whose expression values were roughly consistent across samples. Details on data generation and preprocessing are provided in Supplements 1, 2. Preprocessed RNA-Seq data are available upon request to corresponding authors.

### Steps 3 and 4. Gene co-expression network construction and module identification

We used an iterative procedure to identify the module predictors for association testing with depression severity. We first built a co-expression network by calculating the correlation of the pairwise gene expression, applied a hard threshold to the network and then computed the network’s topological overlap matrix (TOM) dissimilarity between the genes (Fig. 1, Step 3). We used the new TOM distance matrix from the hard threshold to construct a hierarchical tree and used dynamic tree cutting to construct modules (Step 4). Steps 3 and 4 (Fig. 1) were repeated for a grid of hard thresholds to obtain a final hard threshold (0.2) that yields similar module sizes (mean size of 200 genes^28^). In the optimal weighted network, we removed edges with correlation values below a threshold of 0.2. Our motivation was to find cluster sizes that were relatively similar in size and with a large enough number of genes for ssGSEA to be effective. Gene set enrichment often tests modules of size 200 genes, and having similar module sizes (Fig. S3) help alleviate potential module-size bias in ssGSEA. We note that we did not detect correlation between module-size and statistical significance of ssGSEA module associations with depression phenotype. We obtained 23 collections of genes (modules) with similar connectivity in the co-expression network. Slight variation of the hard threshold value does not have a meaningful effect on the number of modules.

To perform the hierarchical clustering, we used unsigned weighted correlation networks analysis^20^ (WGCNA), which has been used in a variety of fields (e.g., cancer and brain imaging analysis^29^). Specifically, we measured dissimilarity between pairs of expression values, created a dendrogram of genes, and identified modules from the different levels of similarity structure. We used a dynamic tree cut clustering method^30^ to identify modules from the TOM matrix of this network of normalized gene expression values. The WGCNA tool includes a dynamic tree cut method that provides a flexible dendrogram cutting mechanism that is effective at detecting nested modules.

### Step 5. Projection of module gene sets onto lower-dimensional feature space

We generated normalized enrichment profiles for each gene cluster using single-sample gene set enrichment analysis (ssGSEA)^21,22^. Similar to the notion of eigengene in WGCNA^20^, ssGSEA calculates an enrichment profile of modules in each subject based on individual expression values in the modules. However, instead of using a principal component analysis, ssGSEA is based on the cumulative distribution of the ranked expression values. Particularly, ssGSEA assigns a sample-level enrichment score to a gene module by rank-normalizing the expression values and comparing the empirical cumulative distribution of these ranks inside and outside that module. The scaled module’s score of a sample represents the degree to which its genes are coordinately up- or downregulated within that sample. Consequently, within a particular sample, the expression profile in the higher-dimensional space of genes is projected onto a lower-dimensional space of modules, yielding a smaller set of new variables that helps reduces the hypothesis space’s dimension and is more biologically interpretable.

One of our goals was to test module hypotheses in an independent data set. We felt the ssGSEA would provide a more reproducible mechanism than eigengene for collapsing a set of genes onto a predictor variable. The ssGSEA method can be applied to an independent expression data set directly without recomputing a correlation matrix and the eigengenes. Furthermore, we did not strictly follow the WGCNA protocol to create modules. Rather than optimize a soft threshold power based on the degree of distribution, we optimized a hard threshold cutoff based on similar module sizes.

### Step 6. Testing gene module features for association with depression severity

The modules’ enrichment profiles were then considered as explanatory variables in the linear models to estimate each participant’s scaled MADRS score. In addition to these enrichment scores, the design matrix also included sex, age, BMI, and batch as covariates. Further, because smoking status is known to be associated with depression^31^ and may confound gene expression^32,33^, we also considered an additional model where smoking status is added as a covariate. Moreover, although the majority of participants are Caucasian, the data set also contains other self-reported ancestries (SRAs). As a sensitivity analysis, we tested our hypotheses on a subset of the data with only Caucasian participants to examine whether the results were confounded by multiple SRAs. The *p* values obtained from the analysis are corrected based on Benjamini–Hochberg’s procedure^34^. Genes in modules with false discovery rate (FDR) adjusted *p* values <0.05 were designated as differentially expressed in aggregate. We also search for the enriched genes among the genes within each significant module using GeneAnalytics^35^ and VarElect^36^ of the GeneCard Suites (http://www.genecards.org/) for additional interpretation.

### Network centrality and individual gene’s importance in discriminating phenotypes

In a secondary univariate analysis, we investigated the individual effect of genes by conducting logistic regressions of the diagnosis phenotype (MDD/HC) on each of the 5912 genes and adjusting the *p* values according to the Benjamini–Hochberg procedure^34^. We also computed eigenvector centrality for each of the 5912 genes based on the co-expression network. Centrality analysis approximates the relative importance of genes based on their connectivity within the network structure: a gene with higher centrality is more influential than a low-centrality gene. The simplest centrality metric is degree centrality, which counts the number of connections a specific gene has with other genes. In this analysis, we computed the eigenvector centrality, a variant of degree centrality that takes into account the importance of neighboring genes. Within the most significant modules, we then examined the relationship between each gene’s centrality and its individual importance, measured as the negative log of its adjusted *p* value:

$$s_i = - \log \left( {p_i} \right) \cdot$$

We find that the centralities of genes within statistically significant modules are more correlated with their univariate diagnosis statistical association compared to genes in other modules.

## Results

According to Chi-square test or *t*-test, there was no difference in age, sex, SRA, occupational status, and educational status between the MDD and HC groups (Table 1). The MDD group showed significantly higher BMI and, as expected, more severe depressive symptoms (based on MADRS score) than the HC group. Therefore, even though BMI does not significantly correlate with MADRS (*p* = 0.123), we controlled for BMI in our subsequent analyses to ensure our results were not confounded by BMI.

**Table 1:** Characteristics of the sample

Variable	All (*N* = 157)	MDD (*n* = 78)	HC (*n* = 79)	*t* or *χ*^2^
Age (years)	32 (1)	33 (10)	31 (10)	1.40 (155)
Sex				2.93
(Female/male)	91/66	51/27	40/39	(1)
SRA				
Caucasian	120	59	61	1.09 (5)
African-American	12	6	6	
Native American	4	3	1	
Native Hawaiian/Pacific Islander	2	1	1	
Asian American	4	2	2	
Other	15	7	8	
Occupational status				
Employed full time	55	25	30	7.62 (7)
Employed part time	19	11	8	
Homemaker	5	1	4	
Full-time student	34	11	23	
Unemployed less than 6 months, but expects to work	7	4	3	
Unemployed 6 months or more, but expects to work	1	1	0	
Unemployed 6 months or more and does not expect to work	1	1	0	
Other	2	1	1	
Educational status				
Some high school	3	1	2	10.39 (5)
High school graduate	11	8	3	
Some college/technical school	62	32	30	
College graduate	37	12	25	
Masters or above	10	2	8	
Other	1	0	1	
Smoking status				
Non-smoker	111	46	65	4.52* (1)
Smoker	14	10	4	
BMI	28.1 (6.43)	29.3 (6.81)	26.9 (5.85)	2.33* (155)
MADRS	11.7 (11.76)	22.2 (7.99)	1.8 (2.45)	21.49** (150)

Values enclosed in the parenthesis represent standard deviations (under “All,” “MDD,” and “HC”) or degrees of freedom (under “*t* or *χ*^2^”). The variables of SRA, occupational status, and educational status contained missing values

*HC* healthy controls, *SRA* self-reported ancestry, *BMI* body mass index, *MADRS* total score on Montgomery-Åsberg Depression Rating Scale

*p < 0.05; **p < 0.01

Figure 1 shows the overall workflow for RNA-Seq data analyses. Out of 19,968 identified genes 12,049 genes with a low count were removed. The remaining 7919 significant counts are then normalized and used as inputs to the angle-based outlier (ABO) analysis, an outlier detection method that is robust for high-dimensional data (Supplement 2-ii). We also removed two outlier samples (one MDD and one HC) with distinctly small ABO factors (<0.001, Fig. S1). We note that, if included in the analyses, these two samples would have had exceptionally high TMM normalization factors. In addition, 2007 expression values with high variability (calculated by coefficient of variation) were also filtered out. As a result, input to the gene co-expression network construction is a logCPM matrix of dimension 157 samples × 5912 genes. We note that the size of this filtered data set is similar to that of other gene expression studies^8,28,37^. Our iterative thresholding and dynamic branch cut of the co-expression network (Steps 3 and 4, Fig. 1) results in 23 modules. The number of genes in each module ranges from 86 to 746 genes.

After correcting for multiple hypothesis testing, we find that two modules’ enrichment profiles, DGM-17 (*β* = 15.4, se = 4.21, *p*_raw_ = 0.000352) and DGM-5 (*β* = -5.31, se = 1.75, *p*_raw_ = 0.00284), are significantly associated with MADRS score (Fig. S4). When tested on only 120 samples of Caucasian subjects, despite the reduction in power, these two modules remain significantly associated with this measure of depression severity (*p*_raw_ = 0.0024 and 0.0032). We concluded that the results were robust to SRA. Furthermore, when smoking status was added as a covariate in the linear model, DGM-17 (*p*_raw_ = 0.000395) and DGM-5 (*p*_raw_ = 0.00244) still showed statistically significant associations with MADRS. The results are similar likely because no participants in the data set were heavy smokers. However, because the Bayesian Information Criterion of this model is larger than that of the original model, we presented the result from the original model without smoking status.

**Fig. 2:**
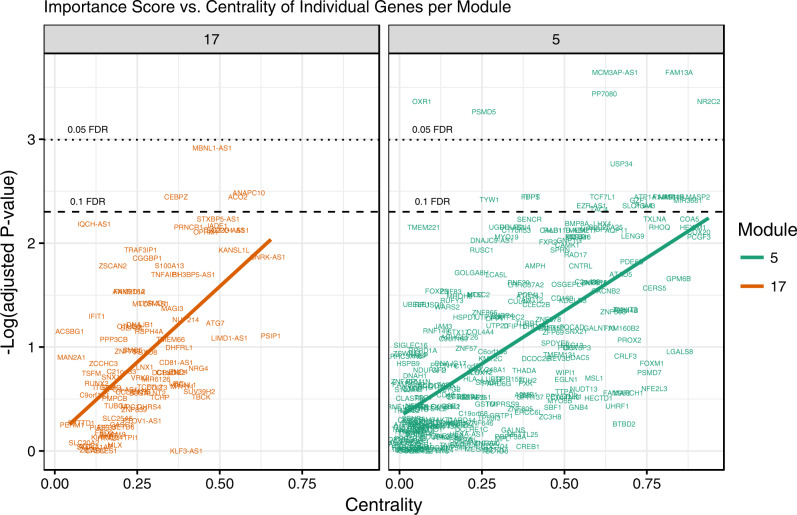
Plot of individual importance vs. eigenvector centrality of genes in DGM-17 and DGM-5. “LOC” genes are not shown. Significant correlation observed between genes’ individual phenotypic and network importance. *R*^2^_(DGM-17)_ = 0.3421; *R*^2^_(DGM-5)_ = 0.3782

DGM-17 and DGM-5 contain 109 and 291 genes, respectively, including VRK2, OPRM1, and TCF7L2 in DGM-17; AKT1, CREB1, CALB1, FAS, FKBP4, FOXP3, HDAC5, and PDE6C in DGM-5, which, as we discuss below, are components of pathways potentially related to mood disorders. Comparing this approach to the traditional individual significance of genes on diagnosis phenotypes, we found DGM-17 and DGM-5 contain significantly more top genes compared to other modules (hypergeometric test results of observing *x*_*i*_ genes from module *i* in the most 100 significant genes are shown in Table S2). Moreover, within these two significant modules, genes’ global centralities are positively correlated with its statistical association with diagnostic status (Fig. 2). This high correlation between univariate gene significance and network centrality implies that genes with high centrality in DGM-17 and DGM-5 tend to be highly correlated with diagnosis status.

### Replication in previous RNA-Seq study of MDD

We used the RNA-Seq study by Mostafavi et al.^16^ as a replication set to test for association with MDD of the significant modules, DGM-17 and DGM-5, from our current study. This independent data set consists of RNA-Seq measurements of 15,231 genes in 463 MDD cases and 452 controls. Of the 291 genes in module DGM-5 and 109 genes in module DGM-17 (Supplements 6, 7), we found 238 and 72 genes in Mostafavi’s study that belong to these two modules, respectively. We applied ssGSEA on these genes to obtain the enrichment scores of 915 subjects for the two modules. Because MADRS score is not reported in this independent data set, we alternatively ran a logistic regression of the diagnosis phenotype (MDD/HC) on the module’s enrichment score, including sex, age, and BMI as in the original modular regression. Even though we only found 82% overlap of module DGM-5’s genes with the Mostafavi data set, DGM-5’s enrichment score was shown to be significantly associated with the diagnosis status in this independent data set (*p* value = 0.033). We found no significant association of DGM-17’s enrichment value with the diagnosis phenotype in the replication set. However, this may be explained by the fact that more than a quarter of the genes in the original, already relatively small, module DGM-17 are not present in the replication data set due to the difference in low-abundance filtering thresholds.

## Discussion

We employed a novel combination of approaches to RNA-Seq data obtained from a cohort of depressed and healthy individuals that led to the replication of a depression gene module in a two-stage analysis. Some of these approaches include enforcing similar module sizes to guide co-expression network thresholding and gene set variation analysis to collapse genes onto modular units of analysis to reduce multiple hypothesis testing. Most gene expression studies have used individual genes as the unit of analysis for differential expression between phenotypes.

Module-based analysis is a sensitive technique to detect weak, but coordinated, gene expression changes at a module level. A related limitation of this approach is that summarizing the score for a module to one value, whether by ssGSEA, eigengene or other dimension reduction techniques, results in the loss of information at the single gene level. However, this technique reduces the high dimensionality of the hypothesis space by clustering thousands of genes into a manageable number of modules of interacting genes that may share similar biological functions. In the lower-dimensional variable space, statistical learning methods can be applied to identify gene modules that are significantly associated with depression severity without overfitting. Thus, we argue that a gene-module approach based on expression networks is a useful statistical model of the genetic architecture of complex diseases such as depression, in which multiple interacting homeostatic systems are affected^38^.

The two statistically significant modules (after FDR adjustment) contain candidate genes for MDD and related disorders. Several genes in module DGM-5 include HDAC5 and CREB1 whose expression has been reported to be altered in MDD patients^39^. The histone methylation processes in which HDAC5 participates have also been implicated across different psychiatric disorders^40^. Linkage of variation in CREB1, the cyclic AMP response element-binding protein gene, to anger expression and treatment outcome in MDD patients^41,42^ as well as gender-specific susceptibility for MDD^43,44^ has been reported. CREB1 is also considered one of the important targets of antidepressants^45^. FOXP3, an intracellular marker for regulatory T cells (Tregs), has shown decreased expression level in depressed patients compare to HC group^46^, while our group previously reported increased circulating numbers of Tregs in MDD versus HC^47^. FOXP3 also plays an essential role in maintaining homeostasis of the immune system, one of the pathways that have significant association with aggregate psychiatric disorders^40^. Variation in FAS, a gene involved in T cell activation and apoptosis, is associated with antidepressant prognosis^48^. A significant increase in FAS expression is also observed in depressed patients^49^.

Another noteworthy gene in module DGM-17 is OPRM1 because of its association with depression symptoms through interaction with stressful life events^50^. Alteration of opioid neurotransmission has also been observed in MDD patients^51^. Additional gene-level information in the significant modules, DGM-5 and DGM-17, is summarized in Table 2 based on relevance to mood disorders from the literature. We found more mood disorder-related genes in the literature for DGM-5 than DGM-17, which may explain the fact that DGM-5 replicated while DGM-17 did not. However, we acknowledge that many of the genes found in the mood disorder literature have not been well replicated because, in part, MDD is a complex disorder of heterogeneous etiology. This complexity is a potential motivation for modular approaches that accumulate the coordinated variation of genes to detect gene modules related to depression. Module DGM-5 also contains more genes than DGM-17; however, we did not find evidence of module-size bias, finding no correlation between module size and statistical significance of modules (results not shown). Another reason that DGM-17 did not replicate may be due to the lower overlap of genes in the replication data set (83% for DGM-5 vs 73% for DGM-17).

**Table 2:** Mood disorder-related genes in significant modules and summary of their relevance to mood disorders from the literature

Module	Gene	Description/related pathways	Prior studies linkage to mood disorder/schizophrenia
DGM-17	OPRM1	μ-opioid receptor/GABAergic synapse	Stressful life events^50^ Sustained sadness condition in women^51^ Response to antidepressants^65^
DGM-5	HDAC5	Histone deacetylase 5/ phospholipase-C Pathway	MDD pathophysiology^39^ Histone pathways^66^
DGM-5	CREB1	The cyclic AMP response element-binding protein 1, sequence-specific DNA binding and enzyme binding/constitutive signaling by AKT1 E17K in cancer	MDD pathophysiology^39^ Anger expression and treatment outcome in MDD patients^41,42^ Gender-specific susceptibility for MDD^43,44^ Important targets of antidepressants^45^
DGM-5	FOXP3	forkhead box P3, the marker for regulatory T cells/Th2 differentiation pathway	Decreased expression level in depressed patients^46^ Immune system responses^40^
DGM-5	FAS	fas cell surface death receptor, T-cell activation and apoptosis/ bacterial infections in CF airways, allograft rejection	Antidepressant prognosis^48^ Expression increase in depressed patients^49^
DGM-5	FKBP4	FK506 Binding Protein 4, paralog of FKBP5/ PEDF induced signaling, HSF1-dependent transactivation	FKBP5: strong evidence for association with MDD^67–72^
DGM-5	AKT1	AKT serine/threonine kinase 1, critical mediator of growth factor-induced neuronal survival/ ICos-ICosL pathway in T-helper cell, development IGF-1 receptor signaling	Schizophrenia^73–75^ Depression in different populations^76^ Neuronal pathways^66^
DGM-17	VRK2	Vaccinia related Kinase 2/nuclear envelope reassembly, mitotic prophase.	Schizophrenia^77–79^
DGM-17	TCF7L2	Transcription Factor 7 Like 2/Wnt signaling pathway	Schizophrenia^80^ Genetic variants that are crucial in MDD susceptibility^81^

In addition to containing several candidate genes, DGM-5 and DGM-17 show enrichment (*q* value 0.2) for several pathways involving immune function (Table 3). The enrichment of the apoptosis pathway in DGM-5 suggests a genetic signature involving brain region-specific volume reduction due to cell loss in MDD^52,53^. The enriched PI3K/AKT activation pathway is involved in apoptosis and plays a role in mRNA translation of type I interferon-dependent genes^54^. The viral protein R (VPR) pathway, enriched in DGM-17, is involved in the induction of apoptosis in proliferating cells and B cell signaling. The DMG-5 module contains the binding protein for VPR (VPRBP in Supplement 7), which suggests additional overlap of the function of these two modules.

**Table 3:** Reactome pathway enrichment results of the two statistically significant MDD modules DGM-5 (replicated) and DGM-17

REACTOME pathways	Genes in pathway	*p* value	FDR *q* value	Over lapping genes
DGM-5: 291 genes				
Apoptosis	148	1.19e−3	0.108	*AKT1, BAD, PSMD5, PSMD7, FAS*
Downstream signaling by B cell receptor	97	5.76e−4	0.108	*AKT1, BAD, CREB1, PSMD5, PSMD7*
PIP3/AKT and PI3K/AKT signaling activation	29	4.82e−4	0.108	*AKT1, BAD, CREB1*
GAB1 signalosome	38	1.07e−3	0.108	*AKT1, BAD, CREB1*
PI3K events in ERBB4 and ERBB2 signaling	38	1.07e−3	0.108	*AKT1, BAD, CREB1*
tRNA aminoacylation	42	1.44e−3	0.108	*WARS2, DARS2, LARS*
AKT phosphorylates targets in the cytosol	12	1.77e−3	0.108	*AKT1, BAD*
DGM-17: 109 genes				
Interactions of Vpr with host cellular proteins	33	2.38e−5	0.016	*NUP214, SLC25A5, PSIP1*

Comprehensive results of the pathway enrichment analysis for all modules are presented in Table S1. The Reactome enrichment FDR *q* value threshold for DGM-5 and DGM-17 is 0.2

The apoptosis signal in the blood expression may originate from the brain (e.g., neuronal death due to apoptosis) and/or from other sources of cellular stress (e.g., activated T cells). The detection of the apoptosis signal in DGM-5 suggests the signal may be brain derived. One may strengthen the evidence for brain-derived apoptosis by testing for the association of expression of apoptosis genes with brain volumetric variation. This hypothesis could be tested in a whole-brain approach or a more targeted region-of-interest approach, conditioning on MDD status, and adjusting for age.

As a secondary analysis, we compared the co-expression network centrality of each gene with the statistical significance of its univariate effect on MDD status. Within the most significant modules, we found a positive correlation between a gene’s centrality and the statistical significance of the gene’s differential expression. In other words, “hub” genes in these top modules are potentially more predictive of the diagnosis phenotype compared to other genes that have lower centrality in the modules. In addition to the cumulative variation of genes within modules, using information related to the centrality of genes may improve the discovery of MDD-related genes and further limit the number of hypothesis tests. Hub genes in significant modules also may make it easier to identify biologically meaningful genes^55^.

One of the limitations of our study is the relatively small sample size. However, the dimensionality reduction, multiple test adjustment, and replication in a previous RNA-Seq MDD study adds evidence that module DGM-5 is not an artifact. A recent microarray study^56^ did not replicate individual gene effects found in the Mostafavi RNA-Seq study. However, their meta-analysis of the *p* values identified six genes that showed a consistent effect (the genes had *p* < 0.05 in both studies)^56^. Similarly, Leday et al.^57^ used a Bayesian technique to identify concordant gene effects across two independent cohorts. DGM-5, in the current study, contains many genes that are biologically relevant or previously associated with mood disorders (Table 2). However, the pathway enrichment signals of DGM-5, such as apoptosis, point to genes outside of Table 2 as playing an important role in MDD etiology. Incorporating eQTL analysis may fill in part of the functional gaps in the DGM-5 network and further characterize the mechanisms of this mood disorder module.

In the first stage of our analysis, we used MADRS as the primary outcome with the aim of including greater phenotypic variation than the diagnostic phenotype. Using a precision quantitative trait in a population has the advantage of capturing more variation than a case–control phenotype and may also have more power. For example, it has been shown that dichotomizing a trait variable at the median reduces the power by the same amount as throwing away 1/3 of the data^58^. One limitation of treating MADRS quantitatively is the lack of variation in the scale among healthy subjects. In the second stage analysis, we used diagnostic status as the dependent variable because depression severity was not available.

Another potential limitation of our study is the use of gene expression from PBMCs, which contain many cell types and may not detect brain-specific mechanisms^16,56^. Peripheral blood is an easily accessible source of cells that may more easily translate into a clinical biomarker compared to cell or tissue-specific gene expression. We did not find significant overlap between our top module genes and known cell-type signatures; however, depression-associated changes in cell frequencies may account for some of the differences in gene expression^59^. While effect sizes may be diluted if small subsets of cells contribute to the signal, we were able to detect module-level signals that replicated in an independent study. Deconvolution methods may help identify cell-specific differences in major immune system cells (such as monocytes, T­helper, B and NK cells) and uncover cell-specific gene expression changes associated with mood disorder phenotypes^60^.

Our modular approach aggregates the effects of genes with shared variation to discover depression gene modules. This approach is influenced by GWAS studies showing that individual variants with small effect sizes, dispersed throughout the genome, drive complex disease risk by key genes and regulatory pathways^61^. Methods for aggregating genetic variation and association signals from prior biological knowledge have been used in GWAS to facilitate more powerful analysis^62,63^. At the level of gene expression, we aggregate the variation from co-expressed genes into modules. This aggregation was done without prior pathway knowledge and in an unbiased way (not conditioned on the phenotype) to mitigate multiple hypothesis testing. Future studies to refine and characterize these depression-related modules will involve identifying regulatory variants through cis- and trans-eQTL and interaction QTL analysis^64^.

Our analysis used stranded RNA-Seq preprocessing where the forward direction was used for the second fast sequence files. This stranded preprocessing enriches for antisense non-coding RNA, sometimes called Natural Antisense Transcripts (NATs). These NATs are labeled with AS1 (for antisense) appended to their gene symbols, and they are known to recruit epigenetic machinery and other mechanisms to regulate coding RNA (mRNA/genes). In addition to NATs, stranded preprocessing enriches for protein coding genes that can be transcribed in the antisense direction, which occurs for a significant proportion of mammalian genes (i.e., protein coding). Thus, the replicated module (M5) contains genes that are enriched for antisense expression of protein coding genes and expression of NATs that regulate partner coding genes through an antisense mechanism. We include the RNA-Seq data preprocessed for both antisense RNA and sense RNA gene expression in the github repository (https://github.com/insilico/DepressionGeneModules).

## Electronic supplementary material

Supplementary Material

## Data Availability

https://github.com/insilico/DepressionGeneModules
