# Correction: Identification and replication of RNA-Seq gene network modules associated with depression severity

**DOI:** 10.1038/s41398-020-00968-2

**Published:** 2020-08-12

**Authors:** Trang T. Le, Jonathan Savitz, Hideo Suzuki, Masaya Misaki, T. Kent Teague, Bill C. White, Julie H. Marino, Graham Wiley, Patrick M. Gaffney, Wayne C. Drevets, Brett A. McKinney, Jerzy Bodurka

**Affiliations:** 1 Department of Mathematics, The University of Tulsa, Tulsa, OK USA; 2 Laureate Institute for Brain Research, Tulsa, OK USA; 3 School of Community Medicine, University of Tulsa, Tulsa, OK USA; 4 Department of Educational Psychology, University of Nebraska-Lincoln, Lincoln, NE USA; 5 Departments of Surgery and Psychiatry, University of Oklahoma School of Community Medicine, Tulsa, OK USA; 6 Department of Pharmaceutical Sciences, University of Oklahoma College of Pharmacy, Tulsa, OK USA; 7 Department of Biochemistry and Microbiology, Oklahoma State University Center for the Health Sciences, Tulsa, OK USA; 8 Tandy School of Computer Sciences, The University of Tulsa, Tulsa, OK USA; 9 Department of Surgery, Integrative Immunology Center, University of Oklahoma School of Community Medicine, Tulsa, OK USA; 10 Arthritis and Clinical Immunology Research Program, Division of Genomics and Data Sciences, Oklahoma Medical Research Foundation, Oklahoma City, OK USA; 11 Janssen Research & Development, LLC, Johnson & Johnson, Inc, Titusville, NJ USA; 12 Stephenson School of Biomedical Engineering, University of Oklahoma, Norman, OK USA

**Keywords:** Depression, Predictive markers

Correction to: *Translational Psychiatry* 10.1038/s41398-018-0234-3 published online 5 September 2018

This Article has been corrected to clear up scientific details (see the authors’ summary below).

“Our analysis used stranded RNA-Seq preprocessing where the forward direction was used for the second fast sequence files. This stranded preprocessing enriches for antisense non-coding RNA, sometimes called Natural Antisense Transcripts (NATs). These NATs are labeled with AS1 (for antisense) appended to their gene symbols, and they are known to recruit epigenetic machinery and other mechanisms to regulate coding RNA (mRNA/genes). In addition to NATs, stranded preprocessing enriches for protein coding genes that can be transcribed in the antisense direction, which occurs for a significant proportion of mammalian genes (i.e., protein coding). Thus, the replicated module (M5) contains genes that are enriched for antisense expression of protein coding genes and expression of NATs that regulate partner coding genes through an antisense mechanism. We include the RNA-Seq data preprocessed for both antisense RNA and sense RNA gene expression in the github repository (https://github.com/insilico/DepressionGeneModules).”

