# Supplementary material for: Identification and replication of RNA-Seq gene network modules associated with depression severity
Source: Transl Psychiatry. 2018 Sep 5;8:180. doi: 10.1038/s41398-018-0234-3 (PMC6125582; doi:10.1038/s41398-018-0234-3)
Supplement: Supplementary file 1 — Supplementary Material [file 41398_2018_234_MOESM1_ESM.pdf]

Supplementary materials

"Identification and replication of RNA-Seq gene network modules associated with depression severity" by Trang Le et al.

Supplement 1. RNA-Seq data generation

Morning blood samples were obtained from the participants, and peripheral blood mononuclear cells (PBMC) were isolated using cell preparation tubes. Then, we quantified RNA expression by analyzing complementary DNA derived from the PBMCs with RNA-Seq. RNA was obtained from frozen peripheral blood mononuclear cells (Inflammation and neurological disease-related genes are differentially expressed in depressed patients with mood disorders and correlate with morphometric and functional imaging abnormalities) using Qiagen (Hilden, Germany) Qiashredder columns to homogenize the cell lysates coupled with and Qiagen RNeasy Mini Kits (Hilden, Germany) for total RNA extraction. The RNA isolation procedure included a DNase digestion step as directed by the Qiagen protocol. RNA was frozen and shipped to the Oklahoma Medical Research Foundation (Oklahoma City, OK). Concentration of RNA was ascertained via fluorometric analysis on a Thermo Fisher Qubit fluorometer. Overall quality of RNA was verified using an Agilent Tapestation instrument. Following initial quality control steps, sequencing libraries were generated using the Illumina Truseq Stranded mRNA with library prep kit according to the manufacturers protocol. Briefly, mature mRNA was enriched for via pull down with beads coated with oligo-dT homopolymers. The mRNA molecules were then chemically fragmented and the first strand of cDNA was generated using random primers. Following RNase digestion the second strand of cDNA was generated replacing dTTP in the reaction mix with dUTP. Double stranded cDNA then underwent adenylation of 3' ends following ligation of Illumina-specific adapter sequences. Subsequent PCR enrichment of ligated products further selected for those strands not

incorporating dUTP, leading to strand-specific sequencing libraries. Final libraries for each sample were assayed on the Agilent Tapestation for appropriate size and quantity. These libraries were then pooled in equimolar amounts as ascertained via fluorometric analyses. Final pools were absolutely quantified using qPCR on a Roche LightCycler 480 instrument with Kapa Biosystems Illumina Library Quantification reagents. Sequencing was performed on an Illumina Hiseq 3000 instrument with paired-end 150bp reads. Samples were sequenced to an average depth of 30 million reads and RNA Integrity Number of 8.6 per sample. RNA-Seq measures gene expression by sequencing, yielding the abundance of each transcript present. Gene transcripts were computed from transcriptomic sequencing using the MAP-RSeq bioinformatics pipeline tool[1]. The Illumin HiSeq 2000 (Illumina, San Diego, CA) sequencing reads were aligned to the human genome build 37.1 using TopHat (1.3.3) and Bowtie (0.12.7). Counting genes and mapping the reads to individual exons was carried by HTSeq (0.5.3p3) and BEDTools (2.7.1), respectively. The total number of read counts was obtained per gene from the mRNA expression. Quality control was graphically assessed pre- and post-normalization with minus- vs.-average and box-and-whisker plots. The GC content and gene length adjustments were also evaluated graphically. Normalization of the gene counts was performed with Conditional Quantile Normalization (CQN), which accounts for differences in library size and also adjusts for GC content and gene length[2]. These normalized values were used for subsequent analyses.

Supplement 2. RNA-Seq data preprocessing

Preprocessing steps described below involved: i) removal of transcripts with low counts (threshold defined below) and normalization, ii) outlier detection, iii) batch effect correction, and iv) exclusion of transcripts of which coefficient of variation were larger than 0.8 in order to eliminate genes with inconsistent expression across samples.

i) Low expression gene removal and normalization: Removing low-expressed transcripts is a necessary step because low values can bias the results when certain types of statistical methods are employed[3]. We considered a gene to be reasonably expressed if, in at least 10% of the samples, its transcript had at least 2-7 reads, depending on the library size (e.g. the total number of raw counts in each sample). In other words, since the library size ranged from 220,667 to 675,792 counts, we removed genes with less than ten counts-per-million (CPM) mapped reads in more than 144 samples, where the CPM reads are computed as followed:

$$CPM = \frac{10^6 \times raw\ counts}{library\ size}$$

We then normalized the raw counts with trimmed mean of M-values (TMM)[4] to account for compositional difference between the libraries. TMM method estimates normalization factors between samples and produces relative expression levels across samples. After removing the low expressed genes and performing within- and between-library normalization, we computed a matrix of $\log_2$ counts-per-million (logCPM) as a variance-stabilizing transformation of the current data set. These steps were performed using the "edgeR" Bioconductor package[5].

ii) Outlier detection: We applied an angle-based outlier (ABO) detection[6] to remove samples with exceptionally small ABO factor. Instead of distances, this method compares the divergence of angles

between pairs of data points. ABO detection has shown to be robust in high dimensional data because it skips over distance, a measure whose contrast between nearest points and farthest points converges to 0 as the space's dimensionality increases[7]. Since ABO factors describe the variance in directions of one data point relative to the rest, data points with small ABO factors imply that the remaining data points are clustered in a specific direction and thus show themselves as potential outliers in the data set (Fig. S1).

iii) Batch effect adjustment: Since erroneous modules of genes can be generated if batch effects are not controlled[8], we adjusted for the batch effect with the function "removeBatchEffect" from the R package "limma"[9] (Fig. S2). Nonetheless, as advised by Nygaard[10], we still included batch as a covariate in our downstream regression models. We also note that although the data contain batch effects, the phenotypes were evenly distributed across batches (Figure S2). This balanced batch-phenotype configuration allows batch adjustment to remove most of the variance attributed to batch without affecting between-group variance and hence help increase statistical power[10].

iv) Highly varied expression values filtering: Finally, prior to the network analysis, among transcripts that have significant counts in at least 16 samples, we excluded transcripts with coefficient of variation larger than 0.8 to obtain genes whose expression values were roughly consistent across samples. We reasoned that expression values that differ greatly across subjects are likely due to technical variability[11]. Filtering by coefficient of variation also helps increase power and prevent false discoveries, hence improves the number of differentially expressed genes in downstream identification analysis[12].

Supplement 3. Figure S1. Angle based outlier (ABO) factors of 159 samples. In the high dimensional RNASeq data, potential outliers are shown in red, corresponding to data points with distinctly small ABO factors.

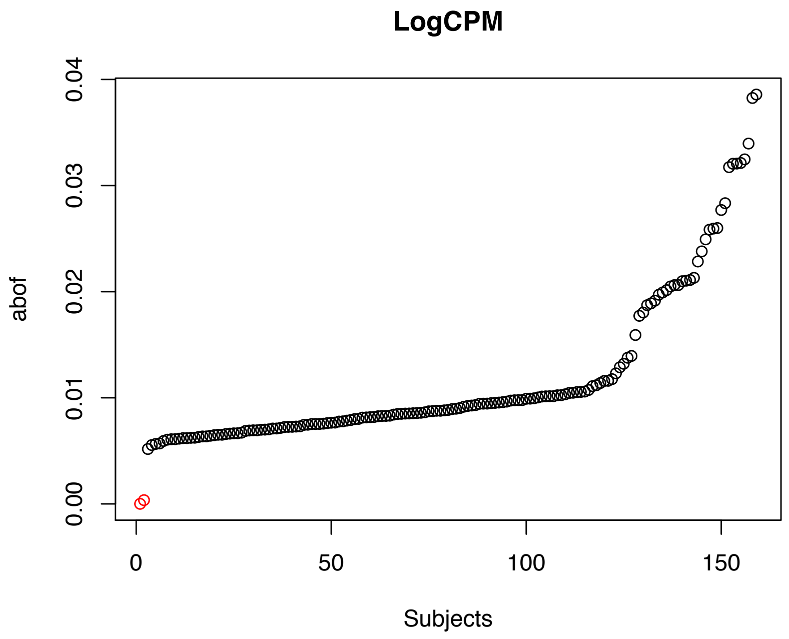

Supplement 4. Figure S2. Two groups of diagnoses equally represented in all batches. The vertical axis (count) represents the number of HC/MDD subjects in each batch.

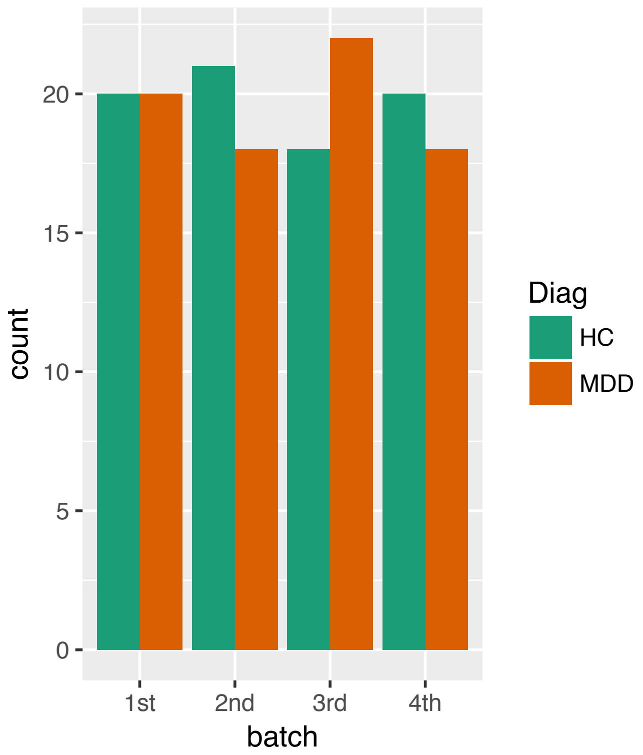

Supplement 5. **Figure S3**. Number of genes in each module.

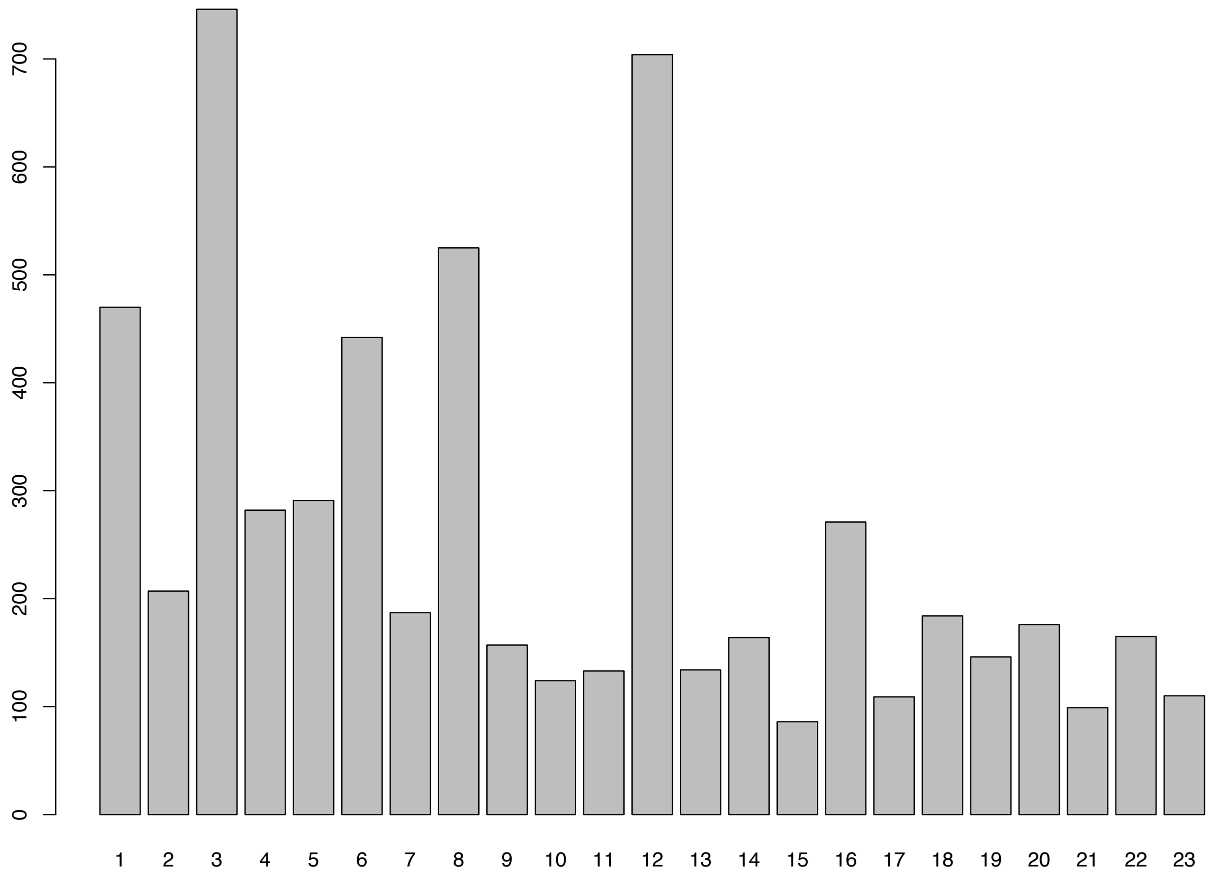

Supplement 6. **Figure S4**. The plot of the modules' composite importance associated with MADRS. At the false discovery rate threshold of 5%, DGM-17 and DGM-5 have significant association with depression severity.

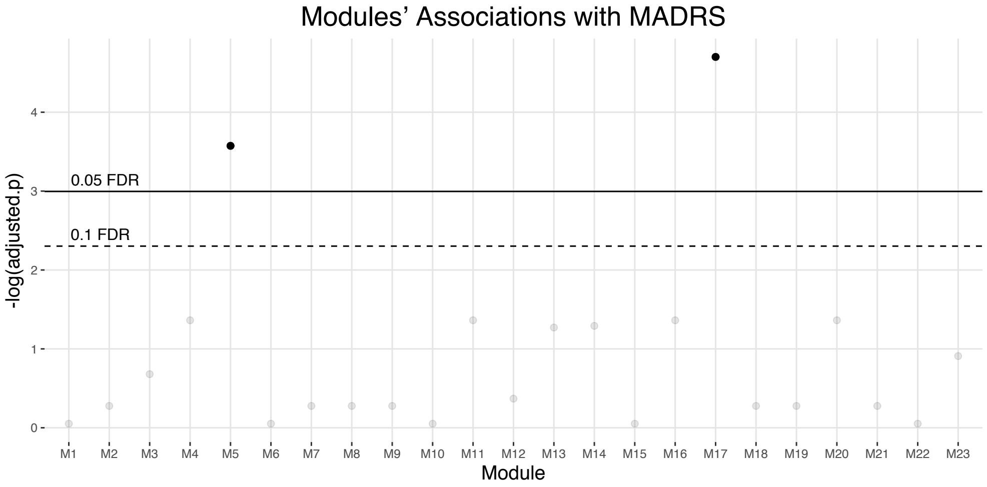

Supplement 7. **Gene list 1.** List of genes in module DGM-5.

A2MP1	CARD14	EVA1C	HEXIM1	MASP2	POLR2J4	SENP1	TUBB1
AFAP1-AS1	CCDC104	EZR-AS1	HLA-J	MCC	PP7080	SIGLEC16	TXLNA
AKAP5	CCDC181	FAM103A1	HMBOX1	MCM3AP-AS1	PPARA	SKIV2L	TYW1
AKT1	CCT6P3	FAM13A	HSPB9	MCTP1	PPM1N	SLAMF1	UBE2E1
ALG12	CD160	FAM160B2	HSPD1	MEGF11	PPP1R3B	SLC13A4	UBTF
ALOX12	CD48	FAM184B	ICMT	METTL25	PPWD1	SLC25A25	UGDH-AS1
ALS2CR12	CD72	FAM86HP	INTS4L2	MIR3661	PRKX	SLC26A2	UHRF1
AMPH	CERS5	FAM98B	ITIH2	MIR635	PROX2	SLC48A1	USP34
ANAPC15	CFD	FAS	JAM3	MLLT10P1	PRPF3	SNRPD2	UTP14A
ANXA6	CLASRP	FDPS	JPX	MMP19	PRPF38A	SNX21	UTP20
AQP11	CLEC2B	FKBP4	KCNIP2-AS1	MPL	PSMD5	SPAG4	VN1R1
ARAP1	CNTRL	FOCAD	KDELR3	MROH6	PSMD7	SPDYE5	VPRBP
ARHGAP27	COA5	FOXM1	KMT2C	MRS2	PTPN14	SPRN	WARS2
ARHGEF26	COL4A4	FOXP3	KRTAP5-1	MSL1	PXK	STX17	WIPI1
ATAD5	COQ6	FTSJ3	LARS	MSTO1	RAB11B-AS1	TAC4	ZC3H8
ATG14	COX20	FXR2	LCA5L	MYO19	RAD17	TACC1	ZFP69
ATP1A1-AS1	CREB1	GALNS	LENG9	MYO5B	RASGEF1A	TAF3	ZNF37BP
ATP2C2	CRLF3	GALNT10	LGALS8	NDUFA2	RBM4B	TBC1D8	ZNF407
ATP5H	CUL4A	GINS3	LHX4	NDUFAF2	REEP5	TCF7L1	ZNF506
B9D2	DARS2	GLE1	LOC100129148	NFE2L3	REV3L	TFIP11	ZNF544
BAD	DCDC2B	GNB4	LOC100379224	NFYC	RHOQ	TFPT	ZNF546
BCS1L	DCLRE1C	GNPTG	LOC100996385	NR2C2	RNF113A	THADA	ZNF552
BMP8A	DDX56	GOLGA8H	LOC101241902	NREP	RNF139-AS1	TM6SF1	ZNF57
BSN	DENND4A	GPM6B	LOC101927275	NT5C2	RNF149	TMEM131	ZNF646
BTBD2	DHODH	GPR137	LOC101927901	NUDT13	RNF224	TMEM139	ZNF790
C11orf42	DNAH1	GPR155	LOC101928063	NUDT5	RNF39	TMEM217	ZNF805
C11orf80	DNAJC17	GPR18	LOC101929767	OSGEPL1	ROCK2	TMEM221	ZNF814
C12orf73	DNAJC9-AS1	GRTP1	LOC102723809	OXR1	RPRD1A	TMEM259	ZNF83
C17orf53	DOT1L	GSE1	LOC102723885	PARD6G	RPUSD3	TMEM263	ZNF865
C19orf68	DPP7	GSTO1	LOC102724246	PCCA	RRP7A	TMPRSS9	ZNF878
C1orf146	DRG1	GTF2F1	LOC646471	PCGF3	RUFY3	TNFAIP8L1	ZNHIT3
C2orf88	ECT2L	GUCY1B2	LOC729732	PCP4L1	RUSC1	TNK1	ZSWIM7
C6orf136	EFR3B	GZF1	LRP11	PDE6C	S100A1	TP53I3	
C9orf169	EGLN1	HACL1	LRRC37A2	PDHX	SAFB	TRAF1	
CACNB2	EML4	HDAC5	LRRC37A6P	PGM3	SBF1	TRIM7	
CALB1	ENTPD3	HECTD1	LYG2	PIGZ	SEC13	TSC1	
CAMK1	ERCC6L	HEXA-AS1	MARCH1	PLEKHB1	SENCR	TTPAL	

Supplement 8. **Gene list 2.** List of genes in module DGM-17.

ACO2	FYTTD1	MAGI3	SMC5
ACSBG1	HIAT1	MAN2A1	SMIM19
ADD3-AS1	HYAL1	MBNL1-AS1	SNRK-AS1
AGA	IFIT1	MIR6126	SNX17
ANAPC10	IPO9-AS1	MLX	SRRD
ANKRD12	IQCH-AS1	MTOR-AS1	STXBP5-AS1
ATG7	ITGAM	MYH11	SUV39H2
B3GALNT2	JADE1	NPC1	TBCK
C1orf122	KANSL1L	NRG4	TCF7L2
C21orf33	KIAA1524	NUP214	TCHP
C9orf163	KLF3-AS1	OPRM1	TMEM66
CABLES1	LIMD1-AS1	OTUD5	TNFAIP1
CASC1	LNX1	PERM1	TPI1
CCDC176	LOC100128494	PIAS3	TRAF3IP1
CCNL1	LOC100130093	PMPCB	TSC22D1-AS1
CD81-AS1	LOC100287944	PPP3CB	TSFM
CEBPZ	LOC100289511	PRNCR1	TUBG1
CGGBP1	LOC100506551	PSIP1	UBC
DCBLD1	LOC101926895	PTOV1-AS1	VRK2
DDIT3	LOC101927151	RSPH4A	YARS
DHFRL1	LOC101927497	RUNX2	ZBTB10
DHRS4	LOC101927770	S100A13	ZCCHC3
DNAJB1	LOC101928243	SERHL	ZNF789
EIF3J	LOC101928371	SETD6	ZNF839
ENO4	LOC101929162	SH3BP5-AS1	ZSCAN2
FAM216A	LOC646719	SLC20A2	
FBXO8	LOC730183	SLC25A5	
FSD2	LYSMD1	SLC4A1AP	

Supplement 9. Table S1. Reactome results of pathways involved in all modules with Reactome-FDR *q*-value < 0.05. Original and adjusted *p*-values are computed from the linear regression of MADRS score on module enrichment profile as described in main paper's Methods section. Modules are ordered by the significance of association with MADRS. Pathways with NA indicates no pathway enrichment found for that gene module.

*For the purpose of exploration, the Reactome's enrichment FDR *q*-value threshold for DGM-5 and DGM-17 is increased to 0.2. The enrichment of the Apoptosis pathway in DGM-5 may suggest a genetic signature involving brain region-specific volume reduction due to cell loss in MDD[13, 14]. The enriched PI3K/AKT activation pathway is also involved in apoptosis and plays a role in mRNA translation of type I interferon-dependent genes[15].

	Module	*p*-value	*p*.adjust	# genes	Pathways involved
1	**DGM-17***	**6.16-05**	**0.002**	**109**	• Interactions of Vpr with host cellular proteins
2	**DGM-5***	**0.002**	**0.016**	**291**	• Apoptosis<br>• Signaling by B Cell Receptor<br>• PIP3/AKT and PI3K/AKT signaling activation<br>• GAB1 signalosome<br>• PI3K events in ERBB4 and ERBB2 signaling<br>• tRNA Aminoacylation<br>• AKT phosphorylates targets in the cytosol
3	DGM-16	0.011	0.085	271	• Metabolism of lipids and lipoproteins<br>• Immune System<br>• Phospholipid metabolism<br>• Developmental Biology
4	DGM-4	0.024	0.134	282	NA
5	DGM-20	0.029	0.134	176	• The citric acid (TCA) cycle and respiratory electron transport<br>• Formation of transcription-coupled NER (TC-NER) repair complex
6	DGM-23	0.039	0.143	110	• Activation of the AP-1 family of transcription factors<br>• DSCAM interactions
7	DGM-13	0.044	0.143	134	• Axon guidance<br>• Developmental Biology
8	DGM-11	0.063	0.181	133	NA
9	DGM-14	0.093	0.234	164	• NFkB and MAP kinases activation mediated by TLR4 signaling repertoire<br>• TRIF mediated TLR3 signaling<br>• TRAF6 mediated induction of NFkB and MAP kinases upon TLR7/8 or 9 activation<br>• MyD88:Mal cascade initiated on plasma membrane<br>• Activated TLR4 signaling<br>• Fatty acid, triacylglycerol, and ketone body metabolism<br>• Metabolism of lipids and lipoproteins<br>• Toll Receptor Cascades<br>• Signaling by NGF<br>• Pre-NOTCH Transcription and Translation
10	DGM-3	0.102	0.234	746	• Adaptive Immune System<br>• Downstream Signaling Events Of B Cell Receptor

					• Immune System<br>• Signaling by the B Cell Receptor (BCR)<br>• Processing of Capped Intronless Pre-mRNA<br>• GAB1 signalosome<br>• Processing of Intronless Pre-mRNAs<br>• Signaling by PDGF<br>• RNA Polymerase II Transcription<br>• Cleavage of Growing Transcript in the Termination Region
11	DGM-12	0.183	0.383	704	• Hemostasis<br>• Platelet activation, signaling and aggregation<br>• Signalling by NGF<br>• Metabolism of proteins<br>• G alpha (12/13) signalling events<br>• Integration of energy metabolism<br>• Platelet homeostasis<br>• Pre-NOTCH Expression and Processing<br>• p75 NTR receptor-mediated signalling<br>• Signaling by Rho GTPases
12	DGM-7	0.262	0.503	187	NA
13	DGM-8	0.365	0.646	525	• mRNA Splicing<br>• mRNA Processing<br>• Immune System<br>• Processing of Capped Intron-Containing Pre-mRNA<br>• Genes involved in Translation<br>• Metabolism of proteins<br>• Metabolism of mRNA<br>• Metabolism of RNA<br>• Adaptive Immune System<br>• 3' -UTR-mediated translational regulation
14	DGM-9	0.469	0.647	157	• RNA Polymerase II Pre-transcription Events<br>• Abortive elongation of HIV-1 transcript in the absence of Tat<br>• MicroRNA (miRNA) Biogenesis<br>• Regulatory RNA pathways<br>• Immune System<br>• Elongation arrest and recovery<br>• Formation of the HIV-1 Early Elongation Complex<br>• RNA Polymerase II Transcription Pre-Initiation And Promoter Opening<br>• Late Phase of HIV Life Cycle<br>• RNA Polymerase II Transcription
15	DGM-22	0.483	0.647	165	• Developmental Biology<br>• Transcriptional Regulation of White Adipocyte Differentiation<br>• Immune System
16	DGM-19	0.515	0.647	146	• Immune System<br>• Myogenesis<br>• Adaptive Immune System<br>• Metabolism of carbohydrates<br>• Class I MHC mediated antigen processing & presentation

					• Inflammasomes<br>• Signaling by TGF-beta Receptor Complex<br>• Platelet activation, signaling and aggregation<br>• Antigen processing-Cross presentation<br>• Response to elevated platelet cytosolic Ca2+
17	DGM-21	0.520	0.647	99	NA
18	DGM-6	0.523	0.647	442	• Immune System<br>• Metabolism of RNA<br>• Metabolism of mRNA<br>• Metabolism of proteins<br>• Influenza Life Cycle<br>• Adaptive Immune System<br>• Influenza Viral RNA Transcription and Replication<br>• Interleukin-2 signaling<br>• Hemostasis<br>• HIV Infection
19	DGM-18	0.534	0.647	184	NA
20	DGM-2	0.579	0.666	207	• Signalling by NGF<br>• SLC-mediated transmembrane transport<br>• PKB-mediated events<br>• Interactions of Vpr with host cellular proteins<br>• p75 NTR receptor-mediated signalling
21	DGM-1	0.639	0.700	470	• Adaptive Immune System<br>• Immune System<br>• Cell Cycle, Mitotic<br>• MHC class II antigen presentation<br>• Cytokine Signaling in Immune system<br>• Cell Cycle<br>• Developmental Biology<br>• DNA Replication<br>• Interferon Signaling<br>• Class I MHC mediated antigen processing & presentation
22	DGM-15	0.705	0.737	86	NA
23	DGM-10	0.948	0.948	124	NA

Supplement 10. **Table S2.** Main effect enrichment of genes in each module. The module dimensionality reduction or feature selection approach is effective at identifying modules that are enriched for individual genes that are statistically significant. The last column gives the probability of observing at least x_i genes from module i in the 100 most significant genes (based on p-values of the logistic regression on clinical phenotype as described in main text) assuming a hypergeometric distribution, taking into account the number of genes in module i. These probability values correlate with the MADRS-significant p-value of each module shown in Supplement 8 (r = 0.9).

DGM- (i)	x_i (# sig. genes)	$P(X \geq x_i)$
**5**	**26**	**8.62e-13**
16	17	2.25e-06
**17**	**8**	**4.70e-04**
13	8	1.82e-03
20	6	7.69e-02
4	8	1.03e-01
23	3	2.85e-01
14	3	5.28e-01
9	2	7.50e-01
12	9	8.57e-01
3	7	9.76e-01
1	2	9.98e-01
6	1	1.00e+00
2	0	1.00e+00
7	0	1.00e+00
8	0	1.00e+00
10	0	1.00e+00
11	0	1.00e+00
15	0	1.00e+00
18	0	1.00e+00
19	0	1.00e+00
21	0	1.00e+00
22	0	1.00e+00

Supplement 11. Gene list 3. Top 100 individual genes with statistically significant association with the diagnostic phenotype (MDD/HC).

FAM13A	LOC100506314	EFCAB14-AS1	WDR90	PAXBP1
MCM3AP-AS1	FGD1	PSMB7	CCDC65	CP
PP7080	AP1G2	RNF167	LOC101926895	LRRN2
NR2C2	MRPS25	RGL4	TYW1	MAK
OXR1	SDCBP2-AS1	C1RL-AS1	FAM168A	GJD3
PSMD5	KIAA1656	MYCBP2-AS1	SLC9A7	SPTY2D1-AS1
CHKA	HBP1	AGAP2	GZF1	COA5
XRCC3	FAN1	ACO2	MIR3661	NOP16
PEX1	CCNI2	C19orf71	MIR324	ZFP36L1
DGCR9	MIPEPP3	KIAA0100	UCHL5	LOC101926943
NEIL1	PHOSPHO1	PDCD2	RNF219-AS1	SARNP
DND1	STARD9	OXA1L	EZR-AS1	SLC34A3
EIF2D	FAM184B	LOC101928371	MAP3K13	PKD2L2
ATP5J	FDPS	TCF7L1	SLC13A4	PPIB
TMEM140	CEBPZ	LOC100379224	PGM3	TXLNA
MBNL1-AS1	MASP2	TRMT1	TAC4	TAP1
USP34	FOXH1	ATP1A1-AS1	CTSB	STXBP5-AS1
LOC101928243	TFPT	LINC00854	TSSK3	SENCR
ANAPC10	PAXBP1-AS1	JUN	LOC100129931	BMP8A
NSA2	EFCAB10	MMP19	KRTCAP3	MXRA8

Supplement 12. Figure S4. (Extension of main paper's Fig. 3) Relationship between the individual gene's importance score and centrality in each module. The modules are ranked from most important (top left) to least important (bottom right). The exact correlation values are given in Supplement 8.

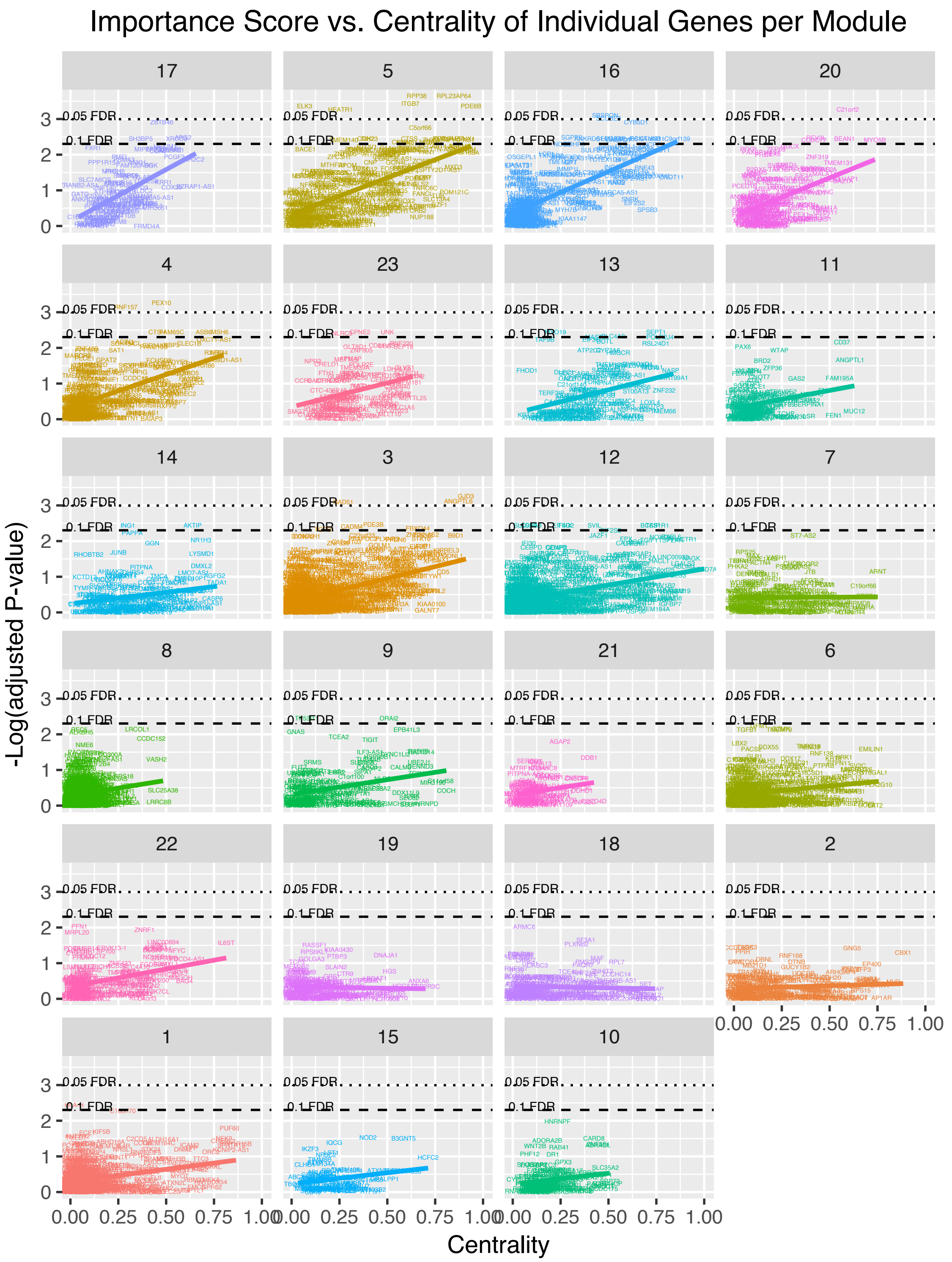

Supplement 13. Table S3. Summary statistics of linear regression models for each module in which the importance scores of genes in the module are regressed on the centrality scores of genes in the module.

DGM-	β coefficients	R^2	p-value
17	0.925	0.137	2.17E-04
5	0.675	0.190	2.44E-14
16	0.944	0.307	2.76E-22
20	0.682	0.093	4.81E-05
4	0.572	0.111	2.58E-08
23	0.364	0.033	0.066
13	0.515	0.085	0.001
11	0.513	0.047	0.015
14	0.315	0.045	0.008
3	0.350	0.064	6.81E-12
12	0.321	0.068	5.75E-12
7	-0.164	0.013	0.123
8	0.069	0.000	0.645
9	0.327	0.058	0.003
21	-0.053	0.001	0.822
6	-0.024	0.000	0.701
22	0.176	0.012	0.170
19	-0.164	0.016	0.126
18	-0.081	0.006	0.321
2	-0.021	0.000	0.757
1	0.298	0.047	2.44E-06
15	0.253	0.015	0.288
10	0.140	0.008	0.338

Supplement 14. Table S4. Interactions between these 14 differential genes and other MDD-related genes.

Our secondary analyses with the conventional logistic regressions of the diagnosis phenotype on individual genes revealed 14 significant effects with FDR threshold of 0.05: MBNL1-AS1, LOC101928243, ANAPC10, LOC101928371, LOC101926895, JADE1, STXBP5-AS1, ADD3-AS1, ACO2, CEBPZ, PRNCR1, FAM13A, MCM3AP-AS1, PP7080, NR2C2, USP34, MMP19, TFPT and TCF7L1. Although no previous MDD associations have been reported for these genes, there is evidence for functional interactions between these genes and known MDD-related genes (www.genecards.org). For example, the XRCC3 gene interacts with the CREB1 gene (discussed above) and FKBP5 gene whose association with MDD has been strongly suggested [16-21]. A comprehensive list of interactions between these 14 differential genes and other MDD-related genes is provided in the Supplementary file InteractingGenes.pdf. We note that an important paralog of FKBP5, FKBP4, participates in module DGM-5. Moreover, several genes in the two modules are associated with schizophrenia, such as the critical mediator of growth factor-induced neuronal survival AKT1 [22-24] (in DGM-5), VRK2 (in DGM-17) which codes for a serine/threonine kinase of the casein kinase I group [25-27], and TCF7L2 (in DGM-17), a component of the Wnt signaling pathway [28]. Our finding of several schizophrenia-related genes in our MDD analysis is not surprising due to the pleiotropy observed across psychiatric disorders, as symptom complexes such as anhedonia and psychosis can be shared across these disorders. Also, markers near AKT1 have been connected to depression in different populations [29], and TCF7L2 contains genetic variants that putatively influence MDD susceptibility [30]. AKT is also a critical mediator of growth factor-induced neuronal survival of which pathways significantly associated with different psychiatric disorders [31].

VarElect - Indirect Results

Prepared for: Trang Le (trang-le+outis@utulsa.edu)
Date: 6/7/2017

Copyright © LifeMap Sciences, Inc.

Results

Implicated Ge Implicating Ge

Implicated Symbol	Implicating Symbol	Description	Category	GIFTs	Matched Phenotypes	Matched Phenotypes	Global Rank (Total Genes	Score (Implicated)	Score (Implicating)
XRCC3	NR3C1	Nuclear Receptor Subfamily 3 Group C Member 1	Protein Coding	76	"major depressive"	1	11	**3.25**	2.48
XRCC3	FKBP5	FK506 Binding Protein 5	Protein Coding	64	"major depressive"	1	4	**3.25**	1
XRCC3	EP300	E1A Binding Protein P300	Protein Coding	74	"major depressive"	1	24	**3.25**	0.69
XRCC3	MTHFR	Methylenetetrahydrofolate Reductase	Protein Coding	66	"major depressive"	1	31	**3.25**	0.64
XRCC3	CREB1	CAMP Responsive Element Binding Protein 1	Protein Coding	72	"major depressive"	1	28	**3.25**	0.31
ACO2	NR3C1	Nuclear Receptor Subfamily 3 Group C Member 1	Protein Coding	76	"major depressive"	1	11	**3.20**	2.39
ACO2	TPH2	Tryptophan Hydroxylase 2	Protein Coding	69	"major depressive"	1	1	**3.20**	1.07
ACO2	POMC	Proopiomelanocortin	Protein Coding	72	"major depressive"	1	14	**3.20**	0.65
ACO2	BDNF	Brain Derived Neurotrophic Factor	Protein Coding	73	"major depressive"	1	6	**3.20**	0.62
ACO2	EP300	E1A Binding Protein P300	Protein Coding	74	"major depressive"	1	24	**3.20**	0.6
NR2C2	NR3C1	Nuclear Receptor Subfamily 3 Group C Member 1	Protein Coding	76	"major depressive"	1	11	**2.98**	1.74
NR2C2	NR1D1	Nuclear Receptor Subfamily 1 Group D Member 1	Protein Coding	69	"major depressive"	1	21	**2.98**	1.5
NR2C2	FKBP5	FK506 Binding Protein 5	Protein Coding	64	"major depressive"	1	4	**2.98**	1.23
NR2C2	EP300	E1A Binding Protein P300	Protein Coding	74	"major depressive"	1	24	**2.98**	1.23
NR2C2	ESR1	Estrogen Receptor 1	Protein Coding	82	"major depressive"	1	52	**2.98**	0.42
TCF7L1	EP300	E1A Binding Protein P300	Protein Coding	74	"major depressive"	1	24	**2.75**	1.81
TCF7L1	GSK3B	Glycogen Synthase Kinase 3 Beta	Protein Coding	74	"major depressive"	1	30	**2.75**	1.06
TCF7L1	HTR2A	5-Hydroxytryptamine Receptor 2A	Protein Coding	69	"major depressive"	1	2	**2.75**	1.03
TCF7L1	BDNF	Brain Derived Neurotrophic Factor	Protein Coding	73	"major depressive"	1	6	**2.75**	0.89
TCF7L1	FKBP5	FK506 Binding Protein 5	Protein Coding	64	"major depressive"	1	4	**2.75**	0.78
ANAPC10	EP300	E1A Binding Protein P300	Protein Coding	74	"major depressive"	1	24	**2.12**	1.41
ANAPC10	BDNF	Brain Derived Neurotrophic Factor	Protein Coding	73	"major depressive"	1	6	**2.12**	0.88
ANAPC10	GSK3B	Glycogen Synthase Kinase 3 Beta	Protein Coding	74	"major depressive"	1	30	**2.12**	0.66

ANAPC10	CREB1	CAMP Responsive Element Binding Protein 1	Protein Coding	72	"major depressive"	1	28	**2.12**	0.58
ANAPC10	PRL	Prolactin	Protein Coding	64	"major depressive"	1	8	**2.12**	0.53
USP34	HTR2A	5-Hydroxytryptamine Receptor 2A	Protein Coding	69	"major depressive"	1	2	**1.64**	1.03
USP34	EP300	E1A Binding Protein P300	Protein Coding	74	"major depressive"	1	24	**1.64**	0.7
USP34	POMC	Proopiomelanocortin	Protein Coding	72	"major depressive"	1	14	**1.64**	0.65
USP34	HTR1A	5-Hydroxytryptamine Receptor 1A	Protein Coding	67	"major depressive"	1	7	**1.64**	0.57
USP34	PRL	Prolactin	Protein Coding	64	"major depressive"	1	8	**1.64**	0.54
FAM13A	HTR2A	5-Hydroxytryptamine Receptor 2A	Protein Coding	69	"major depressive"	1	2	**1.63**	1.09
FAM13A	HTR1A	5-Hydroxytryptamine Receptor 1A	Protein Coding	67	"major depressive"	1	7	**1.63**	0.61
FAM13A	NR3C1	Nuclear Receptor Subfamily 3 Group C Member 1	Protein Coding	76	"major depressive"	1	11	**1.63**	0.53
FAM13A	CRH	Corticotropin Releasing Hormone	Protein Coding	64	"major depressive"	1	12	**1.63**	0.52
FAM13A	CHRM2	Cholinergic Receptor Muscarinic 2	Protein Coding	72	"major depressive"	1	13	**1.63**	0.5
JADE1	EP300	E1A Binding Protein P300	Protein Coding	74	"major depressive"	1	24	**1.40**	1.3
JADE1	CREBBP	CREB Binding Protein	Protein Coding	73	"major depressive"	1	56	**1.40**	0.12
JADE1	PBRM1	Polybromo 1	Protein Coding	65	"major depressive"	1	56	**1.40**	0.1
JADE1	CLOCK	Clock Circadian Regulator	Protein Coding	64	"major depressive"	1	53	**1.40**	0.1
JADE1	AR	Androgen Receptor	Protein Coding	79	"major depressive"	1	54	**1.40**	0.09
CEBPZ	EP300	E1A Binding Protein P300	Protein Coding	74	"major depressive"	1	24	**1.39**	1.08
CEBPZ	GSK3B	Glycogen Synthase Kinase 3 Beta	Protein Coding	74	"major depressive"	1	30	**1.39**	0.42
CEBPZ	TP53	Tumor Protein P53	Protein Coding	79	"major depressive"	1	56	**1.39**	0.3
CEBPZ	CUX1	Cut Like Homeobox 1	Protein Coding	59	"major depressive"	1	56	**1.39**	0.15
CEBPZ	NVL	Nuclear VCP-Like	Protein Coding	55	"major depressive"	1	55	**1.39**	0.09
TFPT	EP300	E1A Binding Protein P300	Protein Coding	74	"major depressive"	1	24	**1.35**	0.84
TFPT	PRL	Prolactin	Protein Coding	64	"major depressive"	1	8	**1.35**	0.58
TFPT	GATA3	GATA Binding Protein 3	Protein Coding	70	"major depressive"	1	24	**1.35**	0.53
TFPT	POMC	Proopiomelanocortin	Protein Coding	72	"major depressive"	1	14	**1.35**	0.5
TFPT	XBP1	X-Box Binding Protein 1	Protein Coding	66	"major depressive"	1	19	**1.35**	0.45
MMP19	BDNF	Brain Derived Neurotrophic Factor	Protein Coding	73	"major depressive"	1	6	**1.24**	0.76
MMP19	HAPLN1	Hyaluronan And Proteoglycan Link Protein 1	Protein Coding	59	"major depressive"	1	24	**1.24**	0.6
MMP19	EP300	E1A Binding Protein P300	Protein Coding	74	"major depressive"	1	24	**1.24**	0.43
MMP19	CREB1	CAMP Responsive Element Binding Protein 1	Protein Coding	72	"major depressive"	1	28	**1.24**	0.38
MMP19	IL10	Interleukin 10	Protein Coding	70	"major depressive"	1	51	**1.24**	0.36

References

1. Kalari KR, Nair AA, Bhavsar JD, O'Brien DR, Davila JI, Bockol MA*, et al*. MAP-RSeq: Mayo Analysis Pipeline for RNA sequencing. *BMC bioinformatics* 2014; **15:** 224.

2. Hansen KD, Irizarry RA, Wu Z. Removing technical variability in RNA-seq data using conditional quantile normalization. *Biostatistics* 2012; **13**(2)**:** 204-216.

3. Bullard JH, Purdom E, Hansen KD, Dudoit S. Evaluation of statistical methods for normalization and differential expression in mRNA-Seq experiments. *BMC Bioinformatics* 2010; **11:** 94.

4. Robinson MD, Oshlack A. A scaling normalization method for differential expression analysis of RNA-seq data. *Genome biology* 2010; **11**(3)**:** R25.

5. Robinson MD, McCarthy DJ, Smyth GK. edgeR: a Bioconductor package for differential expression analysis of digital gene expression data. *Bioinformatics* 2010; **26**(1)**:** 139-140.

6. Kriegel H-P, Schubert M, Zimek A. Angle-Based Outlier Detection in High-dimensional Data. *Proceedings of the 14th ACM SIGKDD international conference on Knowledge discovery and data mining* 2008**:** 444-452.

7. Aggarwal CC, Hinneburg A, Keim DA (2001). On the Surprising Behavior of Distance Metrics in High Dimensional Spaces. In: *Proceedings of the 8th International Conference on Database Theory*. Springer-Verlag. pp 420-434.

8. Leek JT, Storey JD. Capturing heterogeneity in gene expression studies by surrogate variable analysis. *PLoS genetics* 2007; **3**(9)**:** 1724-1735.

9. Ritchie ME, Phipson B, Wu D, Hu Y, Law CW, Shi W*, et al*. limma powers differential expression analyses for RNA-sequencing and microarray studies. *Nucleic Acids Res* 2015; **43**(7)**:** e47.

10. Nygaard V, Rodland EA, Hovig E. Methods that remove batch effects while retaining group differences may lead to exaggerated confidence in downstream analyses. *Biostatistics* 2016; **17**(1)**:** 29-39.

11. McIntyre LM, Lopiano KK, Morse AM, Amin V, Oberg AL, Young LJ*, et al*. RNA-seq: technical variability and sampling. *BMC genomics* 2011; **12:** 293.

12. Hackstadt AJ, Hess AM. Filtering for increased power for microarray data analysis. *BMC Bioinformatics* 2009; **10:** 11.

13. McKinnon MC, Yucel K, Nazarov A, MacQueen GM. A meta-analysis examining clinical predictors of hippocampal volume in patients with major depressive disorder. *J Psychiatry Neurosci* 2009; **34**(1)**:** 41-54.

14. Eilat E, Mendlovic S, Doron A, Zakuth V, Spirer Z. Increased apoptosis in patients with major depression: A preliminary study. *J Immunol* 1999; **163**(1)**:** 533-534.

15. Kaur S, Sassano A, Dolniak B, Joshi S, Majchrzak-Kita B, Baker DP*, et al*. Role of the Akt pathway in mRNA translation of interferon-stimulated genes. *Proceedings of the National Academy of Sciences of the United States of America* 2008; **105**(12)**:** 4808-4813.

16. Tatro ET, Everall IP, Kaul M, Achim CL. Modulation of glucocorticoid receptor nuclear translocation in neurons by immunophilins FKBP51 and FKBP52: implications for major depressive disorder. *Brain research* 2009; **1286:** 1-12.

17. Binder EB, Salyakina D, Lichtner P, Wochnik GM, Ising M, Putz B*, et al*. Polymorphisms in FKBP5 are associated with increased recurrence of depressive episodes and rapid response to antidepressant treatment. *Nat Genet* 2004; **36**(12)**:** 1319-1325.

18. Ellsworth KA, Moon I, Eckloff BW, Fridley BL, Jenkins GD, Batzler A*, et al*. FKBP5 genetic variation: association with selective serotonin reuptake inhibitor treatment outcomes in major depressive disorder. *Pharmacogenetics and genomics* 2013; **23**(3)**:** 156-166.

19. Scheuer S, Ising M, Uhr M, Otto Y, von Klitzing K, Klein AM. FKBP5 polymorphisms moderate the influence of adverse life events on the risk of anxiety and depressive disorders in preschool children. *Journal of psychiatric research* 2016; **72:** 30-36.

20. Lavebratt C, Aberg E, Sjoholm LK, Forsell Y. Variations in FKBP5 and BDNF genes are suggestively associated with depression in a Swedish population-based cohort. *Journal of affective disorders* 2010; **125**(1-3)**:** 249-255.

21. Tatro ET, Everall IP, Masliah E, Hult BJ, Lucero G, Chana G*, et al*. Differential expression of immunophilins FKBP51 and FKBP52 in the frontal cortex of HIV-infected patients with major depressive disorder. *Journal of neuroimmune pharmacology : the official journal of the Society on NeuroImmune Pharmacology* 2009; **4**(2)**:** 218-226.

22. Emamian ES, Hall D, Birnbaum MJ, Karayiorgou M, Gogos JA. Convergent evidence for impaired AKT1-GSK3beta signaling in schizophrenia. *Nat Genet* 2004; **36**(2)**:** 131-137.

23. Mathur A, Law MH, Megson IL, Shaw DJ, Wei J. Genetic association of the AKT1 gene with schizophrenia in a British population. *Psychiatric genetics* 2010; **20**(3)**:** 118-122.

24. Xu MQ, Xing QH, Zheng YL, Li S, Gao JJ, He G*, et al*. Association of AKT1 gene polymorphisms with risk of schizophrenia and with response to antipsychotics in the Chinese population. *The Journal of clinical psychiatry* 2007; **68**(9)**:** 1358-1367.

25. Li M, Wang Y, Zheng XB, Ikeda M, Iwata N, Luo XJ*, et al*. Meta-analysis and brain imaging data support the involvement of VRK2 (rs2312147) in schizophrenia susceptibility. *Schizophrenia research* 2012; **142**(1-3)**:** 200-205.

26. Schizophrenia Working Group of the Psychiatric Genomics C. Biological insights from 108 schizophrenia-associated genetic loci. *Nature* 2014; **511**(7510)**:** 421-427.

27. Zhang B, Gao CY, Zhang HB, Yang B, Yin JF, Wei SG*, et al*. Association of the VRK2 gene rs3732136 polymorphism with schizophrenia in a Northwest Chinese Han population. *Genetics and molecular research : GMR* 2015; **14**(3)**:** 9404-9411.

28. Stefansson H, Ophoff RA, Steinberg S, Andreassen OA, Cichon S, Rujescu D*, et al*. Common variants conferring risk of schizophrenia. *Nature* 2009; **460**(7256)**:** 744-747.

29. Pereira PA, Bicalho MA, de Moraes EN, Malloy-Diniz L, Bozzi IC, Nicolato R*, et al*. Genetic variant of AKT1 and AKTIP associated with late-onset depression in a Brazilian population. *International journal of geriatric psychiatry* 2014; **29**(4)**:** 399-405.

30. Inkster B, Nichols TE, Saemann PG, Auer DP, Holsboer F, Muglia P*, et al*. Pathway-based approaches to imaging genetics association studies: Wnt signaling, GSK3beta substrates and major depression. *NeuroImage* 2010; **53**(3)**:** 908-917.

31. Network, Pathway Analysis Subgroup of the Psychiatric Genomics C. Corrigendum: Psychiatric genome-wide association study analyses implicate neuronal, immune and histone pathways. *Nat Neurosci* 2015; **18**(12)**:** 1861.
